# Optimization of Reflux Conditions for Total Flavonoid and Total Phenolic Extraction and Enhanced Antioxidant Capacity in Pandan (*Pandanus amaryllifolius* Roxb.) Using Response Surface Methodology

**DOI:** 10.1155/2014/523120

**Published:** 2014-07-23

**Authors:** Ali Ghasemzadeh, Hawa Z. E. Jaafar

**Affiliations:** Department of Crop Science, Faculty of Agriculture, University Putra Malaysia, 43400 Serdang, Selangor, Malaysia

## Abstract

Response surface methodology was applied to optimization of the conditions for reflux extraction of Pandan (*Pandanus amaryllifolius* Roxb.) in order to achieve a high content of total flavonoids (TF), total phenolics (TP), and high antioxidant capacity (AC) in the extracts. Central composite experimental design with three factors and three levels was employed to consider the effects of the operation parameters, including the methanol concentration (MC, 40%–80%), extraction temperature (ET, 40–70°C), and liquid-to-solid ratio (LS ratio, 20–40 mL/g) on the properties of the extracts. Response surface plots showed that increasing these operation parameters induced the responses significantly. The TF content and AC could be maximized when the extraction conditions (MC, ET, and LS ratio) were 78.8%, 69.5°C, and 32.4 mL/g, respectively, whereas the TP content was optimal when these variables were 75.1%, 70°C, and 31.8 mL/g, respectively. Under these optimum conditions, the experimental TF and TP content and AC were 1.78, 6.601 mg/g DW, and 87.38%, respectively. The optimized model was validated by a comparison of the predicted and experimental values. The experimental values were found to be in agreement with the predicted values, indicating the suitability of the model for optimizing the conditions for the reflux extraction of Pandan.

## 1. Introduction

Phytochemicals are important compounds found in medicinal plants that exert positive effects on human health or in amelioration of diseases. Though many phytochemicals have been identified, a great many are yet to be identified [1]. According to a report by the World Health Organization, 80% of the population in developing countries depends on traditional medicine for primary health care and 85% of traditional medicine is derived from plant extracts [2]. Extraction and analysis of plant matrices are primary and important processes for the quality control, modernization, and development of herbal formulations [3]. In general, the first step of complete extraction is the selection of plant parts and careful preparation of plant extracts and a thorough review of the existing literature to determine the most suitable protocols for a specific group of phytochemicals or plant species. Traditionally, the extraction of phenolic acids and flavonoid compounds is accomplished by reflux or Soxhlet extraction [4]. However, prolonged extraction at high temperature may degrade flavonoid and phenolic acid compounds [5] and involves a high energy cost. A model for optimizing the most relevant operational parameters is required in order to achieve higher extraction yield. Response surface methodology (RSM) is a collection of statistical and mathematical techniques that is used to optimize the range of variables in various experimental processes with reducing the number of experimental runs, cost, and time compared to other methods [6, 7]. Pandan (*Pandanus amaryllifolius* Roxb.) is a tropical plant of the family Pandanaceae. Pandan has narrow and strap-shaped green leaves with a spiral arrangement [8]. Pandan is characterized by a sweet and delightful flavor and is widely used as a natural flavor in East Asian countries including Indonesia, Thailand, India, and Malaysia. In Malaysia, Pandan leaves are also used in the production of coconut jam, sweets, desserts, and ice cream. A number of studies have demonstrated that Pandan leaves are a valuable source of phenolic compounds [9–11]. However, far too little attention has been paid to optimization of Pandan extract in folk medicine. To the best of our knowledge, there are no studies and reports undertaken to optimize the flavonoid and phenolic extraction from Pandan leaf, following that improving of antioxidant activity using RSM. The aim of this study is to optimize the extraction conditions of a Malaysian* Pandanus amaryllifolius* variety to achieve high TF and TP content and thus to enhance the AC based on the use of RSM method with a central composite design (CCD) for optimization of the reflux extraction conditions.

## 2. Material and Methods

### 2.1. Plant Material

Plant samples (*P. amaryllifolius)* were collected from the North of Malaysia, Bachok, Kelantan province. The samples were identified by the Malaysian Agriculture Research and Development Institute (MARDI) with voucher specimens of MTP008/1. The leaves were shade dried and were powdered using a mechanical grinder. This powered material was used for further analysis.

### 2.2. Extraction

The optimization procedure for the extraction process focusing on the MC (20–80%), ET (30–70°C), and LS ratio (20–40 mL/g) was devised based on three-factor central composite design, as summarized in Table 1. Dissolving of different LS ratio in different MC was designed by RSM software (Table 2) with a total of 20 extraction running sets. Solutions were refluxed at various temperatures (30–70°C) for 2 h. After reflux, the solutions were cooled at room temperature and were filtered with a Whatman number 1 filter paper and used for future analysis.

### 2.3. Determination of Total Flavonoids

After extraction, 1 mL of extracts was diluted with distilled water (4 mL). Initially, NaNO_2_ solution (5%, 0.3 mL) was added to each sample. After 5 min AlCl_3_ solution (10%) and at 6 min NaOH (1.0 M, 2 mL) were added. Absorbance of the solutions was read at 430 nm [12].

### 2.4. Determination of Total Phenolic Content

Leaf extracts (1 mL) were diluted with distilled water (10 mL), and 1 mL of Folin-Ciocalteu reagent was then added. Solutions were allowed to stand for 5 min. Sodium carbonate (2 mL, 20%) was added to the solutions, which were then stored under completely dark conditions at room temperature (25°C) for 90 min. The absorbance of the mixtures was read at 750 nm [13].

### 2.5. Determination of Antioxidant Capacity

#### 2.5.1. * *1,1-Diphenyl-2-picrylhydrazyl (DPPH) Assay

The free radical scavenging activity of extracts was determined according to the Mensor et al. [14] with some modifications. DPPH was dissolved in methanol to give final concentration of 2 mM. Following that, 1 mL of DPPH solution was added to different concentration of Pandan extracts (20, 40, 60, 80, and 100 mg/mL). The mixture was shaken gently and incubated at 28°C in a dark room for 40 min. For the control, methanol was used as a blank. The absorbance of the samples was read at 517 nm using spectrophotometer. BHT (butylhydroxytoluene) and *α*-tocopherol were used as positive controls. The scavenging activity was calculated using the following formula:

(1) %  inhibition =[(absorbance  of  control−absorbance  of  sample)absorbance  of  control]  ×100.

### 2.6. Separation and Analysis of Flavonoids by Ultrahigh Performance Liquid Chromatography (UHPLC)

The UHPLC system (Agilent, Model 1200) with Agilent C18 (4.6 × 250 mm, 5 *μ*m) column was used for flavonoid separation and identification. In this system two mobile phases including 0.03 M orthophosphoric acid (A) and methanol HPLC grade (B) were used. The column temperature, flow rate, and injection volume were adjusted at 35°C, 20 *μ*L, and 1 mL/min, respectively. The range of detecting wavelength was between 260 and 360 nm. Gradient elution was performed as follows: 0–10 min 40–100% B, 10–15 min 100% B, and 15–20 min 100–40% B and finally washing of the column. To prepare the standard solution all flavonoid and phenolic acid standards were dissolved in HPLC grade methanol. The linear regression equation was calculated with *Y* = *aX* ± *b*, where *X* was concentration of flavonoid and *Y* was the peak area of flavonoids obtained from UHPLC [15]. Compounds were tentatively identified by comparison of retention times of standards. All flavonoids and phenolic acids standards were purchased from Sigma-Aldrich (Malaysia).

### 2.7. Experimental Design

RSM was used to determine the optimal extraction conditions for maximizing the TF (*Y*_1_), TP (*Y*_2_), and the AC (*Y*_3_). The central composite experimental design with 3 levels and 3 factors was used to examine the extraction variables. Design-Expert software (Version 7.0.0) was used for data analysis, model building, and experimental design. The statistical significance of the model and model variables was determined at the 5% probability level (*P* < 0.05).

### 2.8. Statistical Analysis

The Design-Expert software (Version 7.0.0) was used for data analysis, model building, and experimental design. Analysis of variance and response surface analysis were employed to determine the regression coefficients and statistical significance of the model terms and to fit the mathematical models of the experimental data that aimed to optimize the overall region for both response variables. A model was applied to predict the response variables as given below:

(2) Y=b0+b1X1+b2X2+b3X3+b12X12+b22X22+b32X32+b1b2X1X2+b1b3X1X3+b2b3X2X3,

where *Y* is the predicted dependent variable, *b*_0_ is a constant that fixes the response at the central point of the experiment, *b*_1_, *b*_2_, and *b*_3_ are the regression coefficients for the linear effect terms, *b*_1_ *b*_2_, *b*_1_ *b*_3_, and *b*_2_ *b*_3_ are the interaction effect terms, and *b*_1_^2^, *b*_2_^2^, and *b*_3_^2^ are the quadratic effect terms, respectively. The relationship between the independent variables (MC: *X*_1_; temperature: *X*_2_, and LS ratio: *X*_3_) and the response variables (TF: *Y*_1_; TP: *Y*_2_, and AC: *Y*_3_) was demonstrated by the response surface plots. Table 1 shows information about extraction temperature, MC, and LS ratio of the 20 experiments.

## 3. Result and Discussion 

### 3.1. Model Fitting, Statistical Significance Analysis, and Response Surface of Reflux Extraction of Total Flavonoid and Phenolics

In this study, extraction temperatures below 80°C were used for the extraction to minimize the possibility of degradation of the flavonoid and phenolic compounds, which has been observed to occur with the application of high temperatures [5, 16]. In addition, the Maillard reaction may occur at high temperatures, resulting in undesired compounds [17]. The results of the experiment and the extraction conditions are shown in Table 2. Significant (*P* < 0.05) regression relationships between the response and independent variables were observed. The TF and TP contents of the extract were more significantly affected by the MC (20–80%), ET (30–70°C), and LS ratio (20–40 mL/g). High TF and TP contents of 1.74 and 6.58 mg/g DW, respectively, were observed in the Pandan extracts using treatment run 5 (Table 2). The predicted TF and TP contents for treatment 5 were 1.7 and 6.55 mg/g DW, which were consistent with the experimental values. The most striking observation from the data is that when the MC (*X*_1_) increased from 20% to 80% (at *X*_2_: 30 and *X*_3_: 40), the TF and TP content increased from 0.71 to 1.44 mg/g DW and from 3.82 to 6.10 mg/g DW, respectively. Increasing the LS ratio resulted in an increment of the TF content of the extracts. In addition, a lower TF content was recorded at lower LS ratio (20 : 1). Increasing the LS ratio from 20 to 40% increased the TF content by 14.4% (at *X*_1_: 80% and *X*_2_: 70°C). In the analysis of the TF content, a good coefficient of determination (*R* ^2^ = 0.98) was obtained, where the model explained most of the observed variations (Table 3). Significant (*P* < 0.05) linear and quadratic effects of the ET, MC, and LS ratio on the TF content (*Y*_1_) were observed. The model *P* value of 0.0001 implies that the model is significant, with only a 0.01% chance that a “model *P* value” this large could be due to noise. The lack of fit test for the model describes the variation in the data around the fitted model. If the model does not fit the data well, the lack of fit value will be significant; consequently, proceeding with investigation and optimization of the fitted response surface is likely to give misleading results. The “lack of fit *P* value” of 0.366 obtained herein implies that the lack of fit is not significant relative to the pure error. However, no interactive effect of the independent variables was observed. The predicted model obtained for TF (*Y*_1_) was as follows:

(3) Y1=+1.26+0.36X1+0.095X2+0.057X3+0.018X1X2+0.023X1X3+0.023X2X3−0.16X12+0.021X22+0.00938X32.

The data in Table 2 demonstrate that when the LS ratio increased from 20 : 1 to 40 : 1, the TP content also increased by about 5.4% (at *X*_1_: 80% and *X*_2_: 70°C). It is plausible that these results are due to the fact that more solvent could enter the cells while more phenolic compounds could permeate into the solvent at higher LS ratios [18]. The results of previous study showed that as the liquid/solid ratio increased, the content of phenolic compounds in the extract of* E. oleracea *was enhanced [19]. The highest content of phenolics was reportedly obtained from fruits of* E. oleracea *at a LS ratio of 40 : 1 (mL/g). The results of another study showed that a high total phenolics value was obtained from* Parkia speciosa* at the LS ratio of 20 mL/g, whereas the total phenolics content was unaffected by the ET [20]. In current study, the predicted model obtained for TP (*Y*_2_) extraction was as follows:

(4) Y2=+5.92+1.15X1+0.12X2+0.097X3+0.030X1X2+0.038X1X3+0.040X2X3−0.76X12+0.026X22−0.10X32.

**Figure 1:**
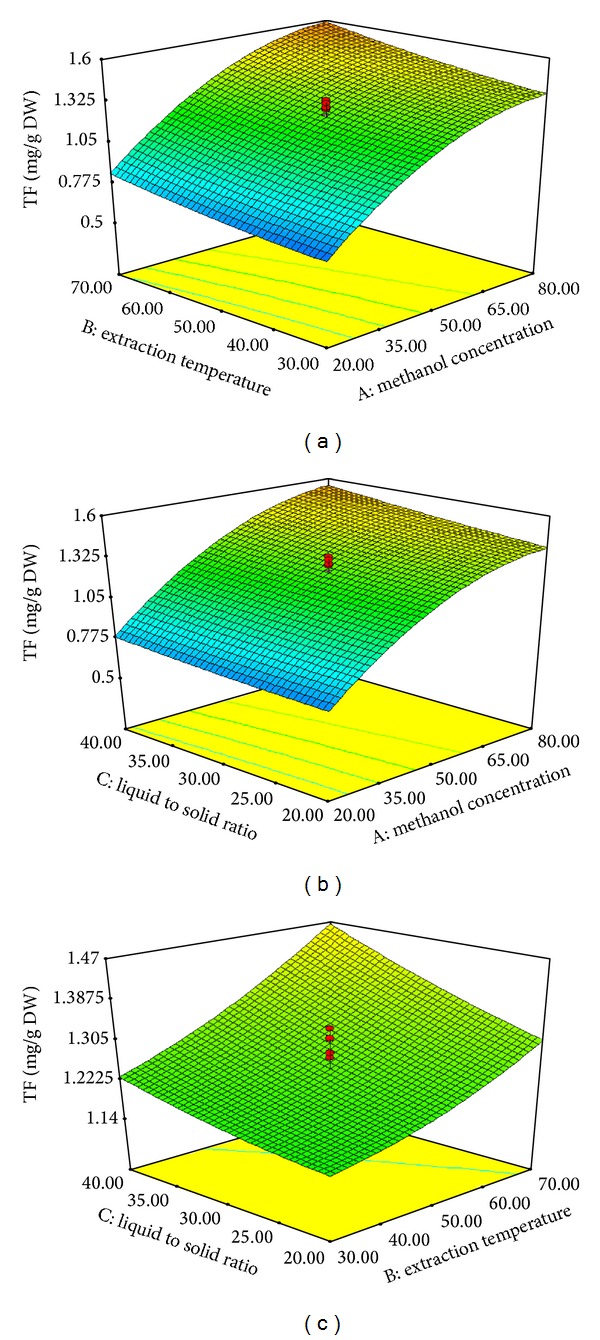
Response surface plots for the effects of MC (20–80%), ET (30–70°C), and LS ratio (20–40 mL/g) on the TF content of Pandan extract. MC and ET (a), MC and LS ratio (b), and ET and LS ratio (c).

**Figure 2:**
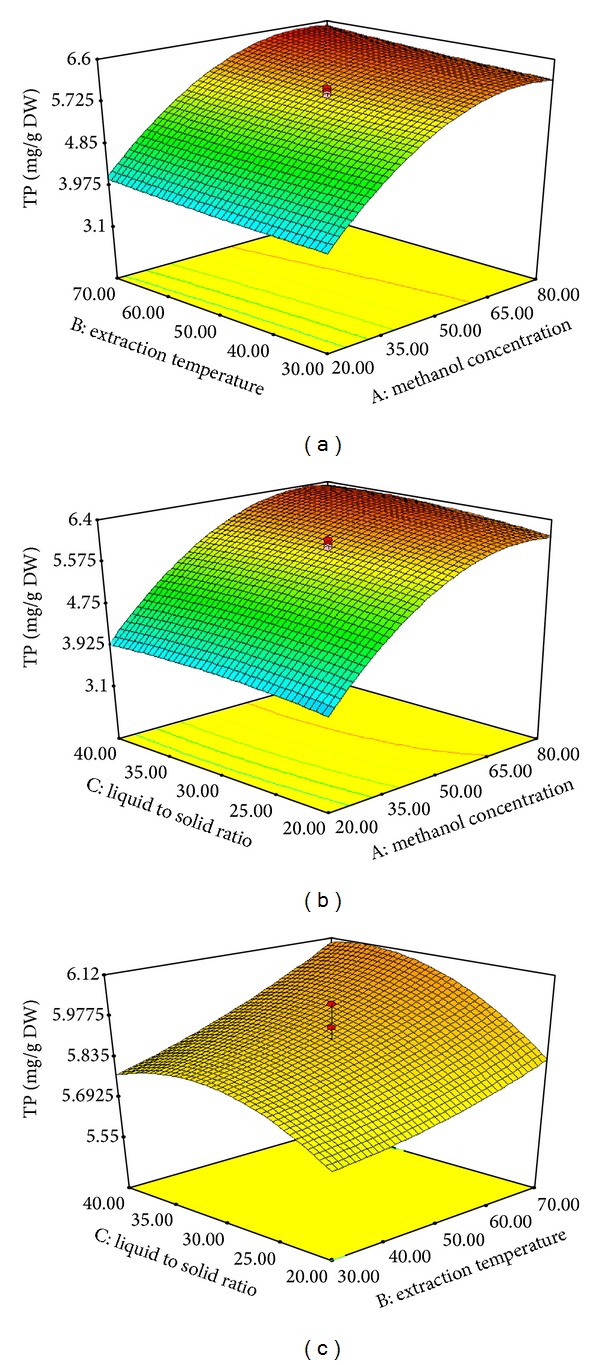
Response surface plots for the effects of MC (20–80%), ET (30–70°C), and LS ratio (20–40 mL/g) on the TP content of Pandan extract. MC and ET (a), MC and LS ratio (b), and ET and LS ratio (c).

The model *P* value of 0.0001 implies that the model is significant (Table 3). There is only a 0.01% chance that such as large “model *P* value” could be due to noise. The “lack of fit *P* value” of 0.295 implies that the lack of fit is not significant, relative to the pure error. The effect of the variables and the interaction of these variables on the responses can be seen in Figures 1 and 2.  Figure 1(a) shows the effect of interaction of the MC and the ET on the TF content of the extract at a fixed LS ratio of 30%. The minimum TF value was obtained at the lowest MC and the maximum TF value was obtained at 80% methanol at the fixed ET of 70°C.  Figure 1(b) shows the effect of the interaction of MC and the LS ratio on the TF content at a fixed ET of 50°C. The minimum TF value was also obtained at the lowest MC and the maximum TF value was obtained at 80% methanol at the fixed LS ratio of 40%.  Figure 1(c) shows the effect of interaction of the ET and the LS ratio on the TF content at a fixed MC of 50%. The minimum TF content was observed at the lowest LS ratio (20%) and the maximum TF value was obtained at a LS ratio of 40% using the fixed ET of 70°C. Moreover, the results indicated that the MC was the most significant factor affecting the responses at the *P* < 0.01 level.  Figure 2(a) shows the effect of interaction of the MC and the ET on the TP content at a fixed LS ratio of 30%. As shown in Figure 2, the TP content increased significantly to ca. 84.5% as the MC increased (from 20 to 80%). Furthermore, the TP content increased slightly as the LS ratio increased from 20 to 40% (Figure 2(b)).  Figure 2(c) shows the effect of interaction of the ET and the LS ratio on the TP content at 50% methanol. The TP content increased significantly as the LS ratio increased up to 32%, but beyond a LS ratio of ~32% the TP content decreased. These findings suggest that the extraction yield of TF and TP was influenced primarily by the MC rather than the ET. It is difficult to explain this result, but this trend might be related to the increased solubility of the flavonoid compounds in the mixture of methanol and water [21]. The findings of the current study are consistent with those of Liyana-Pathirana and Shahidi [22], who found that the TF content of wheat increased with increasing ethanol concentration.

The result of our study showed that TF and TP content increased with increasing of the temperature till 70°C, but we cannot say that this trend will continue even for high temperatures. Previous studies have shown that the application of very high temperatures (⩾95°C) may alter the concentration and composition of phenolic compounds [23]. Some of the authors reported that critical temperature for flavonoids is below 80°C [24]. In different plants and organs this temperature will be variable and according to the previous studies this changes in phenolic acids and flavonoids at high temperature could be related to PAL or CHS enzymes activity at high or low temperature [25]. At high temperature, the flavonoid and phenolic content can be increased as a result of enhancement of their solubility, extraction rate, diffusion rate, and the reduced surface tension and solvent viscosity [26]. However, further increment of the ET may degrade flavonoids and phenolics due to destabilization of the compounds by reaction with other plant components or enzymatic and chemical degradation, thus reducing the extraction efficiency [27]. In contrast with the current results, Gan and Latiff [20] reported that, in the extraction of* Parkia speciosa*, the highest phenolic concentration was achieved at an ET of 35°C and the extraction rate could be increased by reducing the extraction time as well as increasing the ET [28].

### 3.2. Model Fitting, Statistical Significance Analysis, and Response Surface of Reflux Extraction of Antioxidant Capacity (AC)

**Figure 3:**
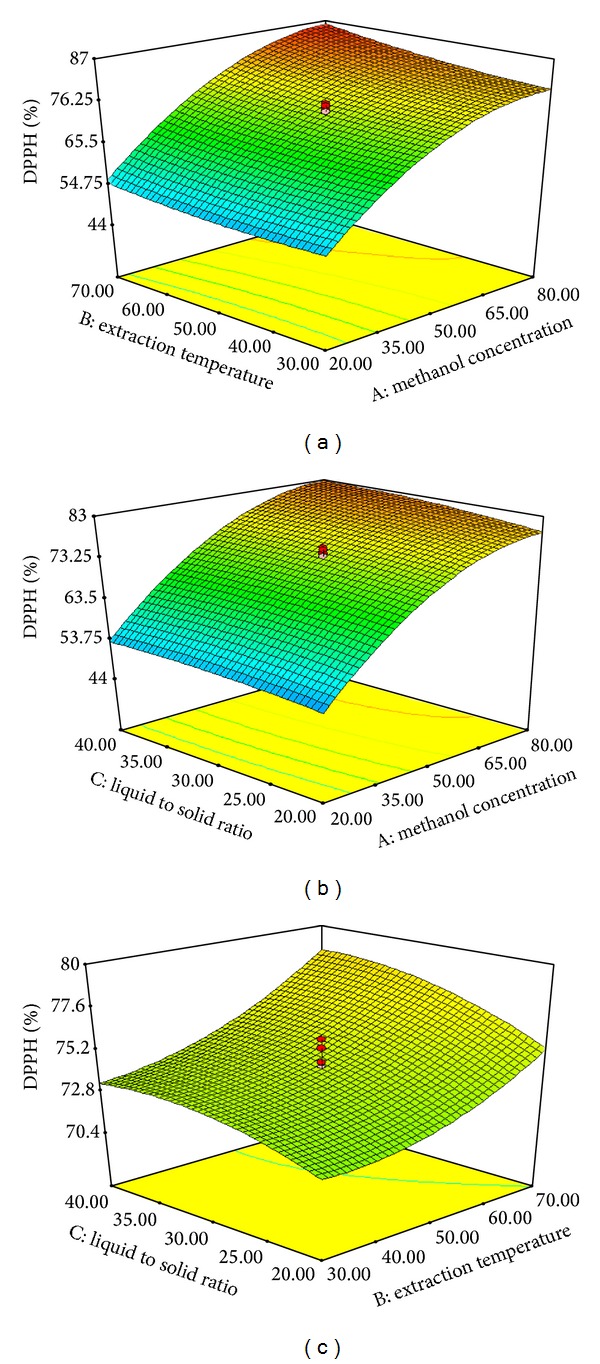
Response surface plots for the effects of MC (20–80%), ET (30–70°C), and LS ratio (20–40 mL/g) on the AC of Pandan extract. MC and ET (a), MC and LS ratio (b), and ET and LS ratio (c).

The AC of the extract was significantly affected by the temperature, solvent concentration, and LS ratio (*P* < 0.05) with three linear effects (*X*_1_, *X*_2_, and *X*_3_), two quadratic effects (*X*_1_^2^ and *X*_2_^2^), and three interactive effects (*X*_1_ *X*_2_, *X*_1_ *X*_3_, and *X*_2_ *X*_3_). The DPPH capacity of the extract ranged from 44.7 to 87.5% when treatments 1 and 5 were, respectively, employed. The predicted AC values for treatments 1 and 5 were, respectively, 44.8 and 87.36%, which were close to the experimental values. The effect of the variables and their interaction on the AC of the Pandan extracts is shown in Figure 3. The AC increased in positive proportion to the MC in the range of 20–80% for the extraction medium. Thus, the MC of the extraction medium had a significant influence on the antioxidant properties of the Pandan extracts. Liyana-Pathirana and Shahidi [22] reported that a higher AC was obtained in the extraction of wheat by using 50% ethanol compared to other aqueous solvents. The current finding is in agreement with those of Pompeu et al. [19] and Kiassos et al. [29], who demonstrated that the concentration of ethanol had a significant influence on the AC of onion extract.

The ET caused a linear increase in the AC of wheat extract, and increasing the temperature increased the total AC. It was confirmed that the rate of extraction of thermally stable antioxidants at elevated temperature was higher than the rate of decomposition of less soluble antioxidants [22]. The temperature utilized during extraction generally influences the compound stability due to chemical and enzymatic degradation and losses by thermal decomposition; these factors have been suggested to be the main mechanisms underlying reduction of the polyphenol content in the extraction of grape [30]. Pompeu et al. [19] obtained high AC in the extraction of* Euterpe oleracea *at a temperature of 58°C. Similarly, high AC of wheat extracts was observed when a temperature of 61°C was utilized [22] and high AC was achieved (83.37%) when the LS ratio was low (20 mL/g). The latter is plausibly due to increased probability of the antioxidant components coming into contact with the extraction solvent as the amount of solvent increased. However, further increase of the LS ratio may dilute the extraction solution thereby lowering the AC. In another study, high AC of* Parkia speciosa *extract was achieved using a liquid/solid ratio of 20 mL/g [20]. The regression equation obtained for the AC (*Y*_3_) as the response variable also showed significant (*P* < 0.05) dependence of *Y*_3_ on the variation of the independent variables. The predicted model obtained for *Y*_3_ is given below:

(5) Y3=+74.46+14.19X1+2.20X2+1.20X3+1.18X1X2+0.40X1X3+0.40X2X3−6.89X12+1.05X22−0.82X32.

The model *P* value of 0.0001 obtained for the AC implies that the model is significant (Table 3). The “lack of fit *P* value” of 0.266 implies that the lack of fit is not significant, relative to the pure error. There is a 26.65% chance that a “lack of fit *F* value” this large could occur due to noise. One question that needs to be asked, however, is why does software take about five or six center points in the design? The reason is also related to the variance of a predicted value. When fitting a response surface we want to estimate the response function in this design region where we are trying to find the optimum. We want the prediction to be reliable throughout the region and especially near the center since we hope the optimum is in the central region. By picking five to six center points, the variance in the middle is approximately the same as the variance at the edge. If we only had one or two center points, then we would have less precision in the middle than we would have at the edge. As we go farther out beyond a distance of 1 in coded units, we get more variance and less precision. What we are trying to do is to balance the precision at the edge of the design relative to the middle.

### 3.3. Optimization of Reflux Extraction Condition for TF and TP Content and AC

**Figure 4:**
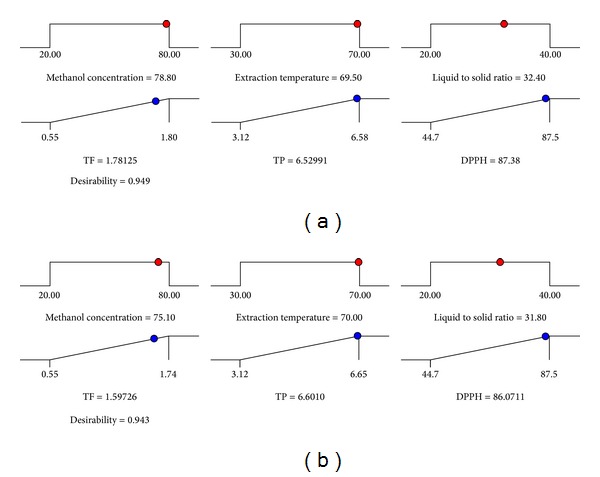
Predicted value of TF, TP, and DPPH activity of Pandan leaf from optimized extraction condition using RSM ((a) optimized condition for TF and DPPH activity; (b) optimized condition for TP).

The optimum reflux extraction conditions for maximizing the TF and TP and for achieving high AC of Pandan extracts were predicted using the Design-Expert software. Multiple graphical and numerical optimizations were carried out to determine the optimum level of independent variables with desirable response goals. Two optimal conditions were developed for the responses: the TF content and AC were maximized using a MC of 78.8%, ET of 69.5°C, and LS ratio of 32.4 mL/g, whereas the corresponding conditions for maximizing TP were 75.1%, 70°C, and 31.8 mL/g, respectively (Table 4, Figure 4).

### 3.4. Verification of the Models

The experiment was performed using the recommended optimum treatment conditions for the three responses to evaluate the adequacy of the response surface models for predicting the optimum response values. As shown in Table 4, the observed values of the TF and TP content and AC were 1.78, 6.601 mg/g DW, and 87.38%, respectively. The response surface models of TF, TP, and antioxidant activity were verified using the experimental and predicted values. The obtained results from verification experiment were in consent with the predicted values, because nonsignificant (*P* > 0.05) difference was observed between the verification experimental and the predicted values (*E*_TF,TP_ = 0.069%; *E*_DPPH_ = 0.48%).

### 3.5. Identification of Flavonoids and Phenolic Acids

**Figure 5:**
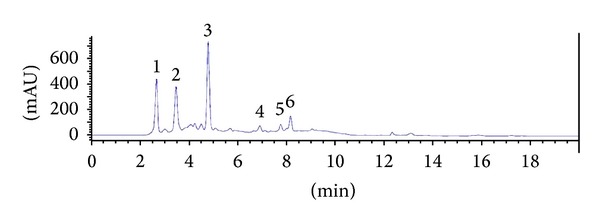
UHPLC chromatogram of Pandan extract. Identified compounds are gallic acid (1), catechin (2), caffeic acid (3), myricetin (4), luteolin (5), and quercetin (6).

Figure 5 shows the UHPLC chromatogram of the identified flavonoid and phenolic acids of the Pandan extract. Gallic acid, (+)-catechin, caffeic acid, myricetin, luteolin, and quercetin were identified in the Pandan extract at concentrations of 0.489, 0.594, 0.856, 0.076, 0.08 and 0.112 mg/g DW, respectively. Caffeic acid was the most abundant compound of the identified compounds, and the concentration of phenolic acids was higher than that of the flavonoid compounds in the Pandan extract.

## 4. Conclusion

The reflux extraction of TF and TP and the AC of Pandan extract were successfully optimized using RSM. The results indicate that the MC, ET, and LS ratio had a significant effect on the TF and TP extraction yields with consequent enhancement of the AC of the extracts. The results can be easily explained on the basis that both the ET and the MC have a positive effect on the solubility of flavonoids in the extraction solution. The most efficient set of reflux conditions for Pandan leaf extraction are at MC 78.8% (*X*_1_), ET 69.5°C (*X*_2_), and LS ratio of 32.4 mL/g (*X*_3_) for maximizing the TF content and AC and 75.1% (*X*_1_), 70°C (*X*_2_), and 31.8 mL/g (*X*_3_) for maximizing the TP content with consequently high AC. Moreover, the models used to fit the response variables were significant (*P* < 0.01), and the “lack of fit” was not significant (*P* > 0.05) for all responses, indicating that the models used to fit the response variables were adequate for representing the relationship between the response values and the independent variables.

## Figures and Tables

**Table 1:** Independent variables and their coded and actual values used for optimization.

Independent variable	Coded level						
	Unit	Symbol	−1	0	+1	Axial (−*α*)	Axial (+*α*)
MC	%	*X*_1_	10.52	50	89.48	20	80
ET	°C	*X*_2_	23.68	50	76.32	30	70
LS ratio	%	*X*_3_	16.84	30	43.16	20	40

MC: methanol concentration; ET: extraction temperature; LS ratio: liquid to solid ratio.

**Table 2:** The result of experimental and predicted values of reflux extraction of TF, TP, and AC.

Run	*X*_1_	*X*_2_	*X*_3_	TF (mg/g DW)		TP (mg/g DW)		AC%	
				Experimental values	Predicted values	Experimental values	Predicted values	Experimental values	Predicted values
1	10.52	50	30	0.55	0.56	3.12	3.18	44.70	44.80
2	80	70	20	1.52	1.50	6.24	6.20	83.20	83.37
3	50	50	16.84	1.16	1.15	5.55	5.50	70.40	70.42
4 (C)	50	50	30	1.33	1.26	5.94	5.90	75.80	74.48
5	80	70	40	1.74	1.70	6.58	6.55	87.50	87.36
6	20	30	20	0.67	0.68	3.79	3.82	52.00	52.19
7	50	76.32	30	1.40	1.39	6.06	6.06	80.00	80.10
8 (C)	50	50	30	1.22	1.26	5.84	5.90	73.00	74.48
9	80	30	40	1.44	1.43	6.10	6.16	79.40	79.81
10	20	30	40	0.71	0.71	3.82	3.85	53.10	52.98
11 (C)	50	50	30	1.31	1.26	5.89	5.90	75.30	74.48
12	50	50	43.16	1.35	1.33	5.95	5.91	75.80	75.60
13 (C)	50	50	30	1.27	1.26	6.02	5.97	74.20	74.48
14	50	23.68	30	1.15	1.17	5.89	5.83	72.70	73.10
15	20	70	20	0.81	0.79	3.99	3.93	53.80	53.43
16 (C)	50	50	30	1.28	1.26	5.94	5.90	74.50	74.48
17	89.48	50	30	1.38	1.40	6.11	6.14	80.50	80.61
18 (C)	50	50	30	1.21	1.26	5.85	5.90	73.80	74.48
19	20	70	40	0.88	0.90	4.10	4.12	54.90	55.82
20	80	30	20	1.37	1.32	6.00	5.98	78.80	77.49

(C): central point; *X*_1_: MC; *X*_2_: ET; *X*_3_: LS ratio.

**Table 3:** Predicted models and statistical parameters calculated after implementation of three-factor central composite design.

Measured parameters	Predicted models	*R* ^ 2^	*R* ^ 2^ (adjusted)	Regression (*P* value)	Lack of fit (*P* value)
TF	+1.26 + 0.36*X*_1_ + 0.095*X*_2_ + 0.057*X*_3_ + 0.018*X*_1_ *X*_2_ + 0.023*X*_1_ *X*_3_ + 0.023*X*_2_ *X*_3_ − 0.16*X*_1_^2^ + 0.021*X*_2_^2^ + 0.00938*X*_3_^2^	0.98	0.97	0.0001	0.366

TP	+5.92 + 1.15*X*_1_ + 0.12*X*_2_ + 0.097*X*_3_ + 0.030*X*_1_ *X*_2_ + 0.038*X*_1_ *X*_3_ + 0.040*X*_2_ *X*_3_ − 0.76*X*_1_^2^ + 0.026*X*_2_^2^ − 0.10*X*_3_^2^	0.99	0.99	0.0001	0.295

DPPH	+74.46 + 14.19*X*_1_ + 2.20*X*_2_ + 1.20*X*_3_ + 1.18*X*_1_ *X*_2_ + 0.40*X*_1_ *X*_3_ + 0.40*X*_2_ *X*_3_ − 6.89*X*_1_^2^ + 1.05*X*_2_^2^ − 0.82*X*_3_^2^	0.99	0.99	0.0001	0.266

**Table 4:** Optimum conditions and experimental value of responses at the optimum conditions.

	Optimum conditions (predicted)	TF content	Optimum conditions (predicted)	TP content	Optimum conditions (predicted)	AC (DPPH assay)
MC (%)	78.8	1.78	75.1	6.601	78.8	87.38
ET (°C)	69.5		70		69.5	
LS ratio (mL/g)	32.4		31.8		32.4	

TF and TP: mg/g DW; AC: %.
